# Disparities of SARS-CoV-2 Nucleoprotein-Specific IgG in Healthcare Workers in East London, UK

**DOI:** 10.3389/fmed.2021.642723

**Published:** 2021-04-27

**Authors:** Naheed Choudhry, Kate Drysdale, Carla Usai, Dean Leighton, Vinay Sonagara, Ruaridh Buchanan, Manreet Nijjar, Sherine Thomas, Mark Hopkins, Teresa Cutino-Moguel, Upkar S. Gill, Graham R. Foster, Patrick T. Kennedy

**Affiliations:** ^1^Centre for Immunobiology, Blizard Institute, Barts and The London School of Medicine and Dentistry, Queen Mary University of London, London, United Kingdom; ^2^Barts Health NHS Trust, The Royal London Hospital, London, United Kingdom; ^3^Barts Health NHS Trust, Newham General Hospital, London, United Kingdom; ^4^Barts Health NHS Trust, Whipps Cross Hospital, London, United Kingdom

**Keywords:** sero-surveillance, healthcare workers, point-of-care, antibody detection, SARS-CoV-2

## Abstract

**Introduction:** SARS-CoV-2 antibody detection serves as an important diagnostic marker for past SARS-CoV-2 infection and is essential to determine the spread of COVID-19, monitor potential COVID-19 long-term effects, and to evaluate possible protection from reinfection. A study was conducted across three hospital sites in a large central London NHS Trust in the UK, to evaluate the prevalence and duration of SARS-CoV-2 IgG antibody positivity in healthcare workers.

**Methods:** A matrix equivalence study consisting of 228 participants was undertaken to evaluate the Abbott Panbio™ COVID-19 IgG/IgM rapid test device. Subsequently, 2001 evaluable healthcare workers (HCW), representing a diverse population, were enrolled in a HCW study between June and August 2020. A plasma sample from each HCW was evaluated using the Abbott Panbio™ COVID-19 IgG/IgM rapid test device, with confirmation of IgG-positive results by the Abbott Architect^TM^ SARS-CoV-2 IgG assay. 545 participants, of whom 399 were antibody positive at enrolment, were followed up at 3 months.

**Results:** The Panbio™ COVID-19 IgG/IgM rapid test device demonstrated a high concordance with laboratory tests. SARS-CoV-2 antibodies were detected in 506 participants (25.3%) at enrolment, with a higher prevalence in COVID-19 frontline (28.3%) than non-frontline (19.9%) staff. At follow-up, 274/399 antibody positive participants (68.7%) retained antibodies; 4/146 participants negative at enrolment (2.7%) had seroconverted. Non-white ethnicity, older age, hypertension and COVID-19 symptoms were independent predictors of higher antibody levels (OR 1.881, 2.422–3.034, 2.128, and 1.869 respectively), based on Architect™ index quartiles; participants in the first three categories also showed a greater antibody persistence at 3 months.

**Conclusion:** The SARS-CoV-2 anti-nucleocapsid IgG positivity rate among healthcare staff was high, declining by 31.3% during the 3-month follow-up interval. Interestingly, the IgG-positive participants with certain risk factors for severe COVID-19 illness (older age, Black or Asian Ethnicity hypertension) demonstrated greater persistence over time when compared to the IgG-positive participants without these risk factors.

## Introduction

Since March 2020, the United Kingdom has enforced three separate restriction policies for its population to limit social interaction and movement in the hope of mitigating the impact of the Coronavirus Disease-19 (COVID-19) pandemic caused by the Severe Acute Respiratory Syndrome Coronavirus 2 (SARS-CoV-2).

As nations globally experienced immense pressure on their healthcare systems, the psychological and economic impacts of the pandemic have been equally challenging. This has resulted in an unprecedented worldwide effort for vaccine development alongside the establishment of robust and rapid diagnostic tests, especially as non-specific early clinical manifestations require accurate diagnosis, ensuring appropriate clinical management, surveillance, and effective control strategies (1, 2).

Serological tests are being developed and evaluated to detect humoral immune responses, specifically immunoglobulins (Ig)G, IgM and total Ig to SARS-CoV-2 (3), to be widely employed across communities irrespective of the presence or absence of symptoms, thus complementing diagnosis outside of the window of positivity for polymerase chain reaction (PCR)-based SARS-CoV-2 test (the gold standard) (4). There are currently two types of antibody tests available: (i) quantitative laboratory tests with antibodies titrated by enzyme-linked immunosorbent assay (ELISA) or Chemiluminescent Microparticle Immunoassay (CMIA), (ii) point-of-care (POC) tests, mainly based on lateral flow chromatographic immunoassays (4, 5), designed primarily to provide easy and relatively inexpensive access to diagnostics.

Lateral flow POC tests for the rapid detection of antibodies can effectively complement PCR diagnosis and antigenic tests for SARS-CoV-2 infection, as IgM and IgG seroconversion occur within 10–12 days and 12–14 days, respectively, after the onset of symptoms (6–9). IgM levels begin to decline by week 5 and almost disappear after week 7, whereas IgG levels persist beyond week 7 (10) reflecting IgG as a more robust indicator of prior exposure (11, 12). Further investigations are required to understand the dynamics of the early humoral immune response to realise the full potential of serological testing for SARS CoV-2.

In this study, we first validate the CE-marked Abbott Panbio™ COVID-19 IgG/IgM Rapid Test Device (Panbio^TM^ test). This *in vitro* diagnostic rapid test (immunochromatographic assay) for the qualitative detection of IgG and IgM antibodies to SARS-CoV-2 nucleocapsid (N) protein, is intended for use in a POC setting and has previously been validated mainly for use with serum and plasma (13). Here we further assess the Panbio^TM^ test for its use with fingerstick capillary and venous whole blood in addition to serum and plasma, which form the matrix equivalence arm (ME) of the study. We then focus on determining the seroprevalence and duration of COVID-IgG and IgM antibodies in healthcare workers (HCWs). Previous studies of COVID-19 patients from across the world (14–17), have shown that HCWs had a 10% greater risk of infection due to the nature of their work and viral exposure to the virus from the hospital setting (18). Our aim was to assess the prevalence of a past immune response to the SARS-CoV-2 virus among HCWs, as measured by detecting seroconversion of SARS-CoV-2-specific IgG and IgM antibodies using the Panbio^TM^ test with confirmation using the Architect™ SARS-CoV-2 IgG test, and to evaluate the persistence of SARS-CoV-2 antibodies at a 3-month follow-up visit. Monitoring HCWs may facilitate early detection of healthcare-associated outbreaks which would allow implementation of management strategies assisting containment (19).

## Materials and Methods

### Population Recruitment

#### Matrix Equivalence Study

Two hundred twenty-eight adults (>18yrs) were recruited over a four-week period from mid-May 2020 after an open invitation was sent locally to the general public living within the Barts Health NHS Trust area, in East London, UK. Individuals known to have had a previous COVID-19 illness (including PCR-confirmed COVID-19) as well as those who were not thought to have been previously exposed to SARS-COV-2, were offered the opportunity to participate. All participants provided informed consent according to the local ethics committee approval (Approved 22/04/2020, South Central - Berkshire Research Ethics Committee ref: 20/SC/0191, ISRCTN60400862) (20).

#### Healthcare Worker Study

Two thousand and fourteen members from the local staff population were recruited during months June—August 2020 from three hospital sites within the Barts Health NHS Trust, in East London, UK. Concordantly with the ME study, individuals with either known or unknown previous exposure to SARS-COV-2 were offered the opportunity to participate, and all participants provided informed consent according to the local ethics committee approval (Approved 29/05/2020, London - Camden & Kings Cross Research Ethics Committee, ref 20/HRA/2675, ISRCTN15634328) (21).

### Study Design

After obtaining written informed consent, study staff verified that each participant met study inclusion and none of the exclusion criteria. The study ISRCTN registrations gives further details of these (20, 21). Demographic information, a brief medical history relating to COVID-19, prior testing results and risk factors, including occupational risk where appropriate, were collected from each participant. Blood samples were then collected.

The ME study aimed to enroll a 1:1 ratio of SAR-Cov-2 positive and negative participants until 103 evaluable positives subjects were enrolled. A total of 228 participants were recruited.

**Figure 1:**
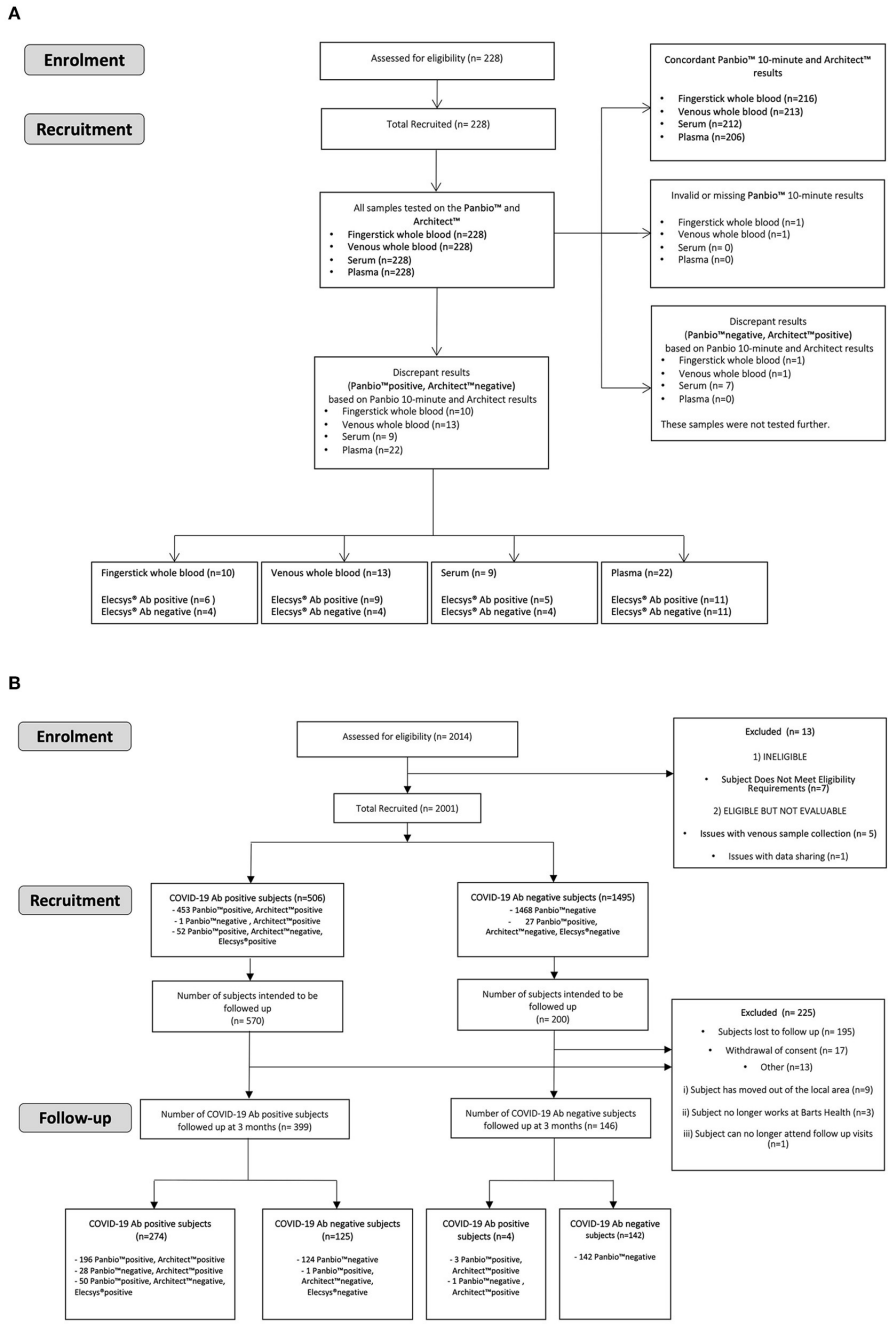
Consort diagram reporting participant flow of: **(A)** ME study and **(B)** HCW study.

For the HCW study, from the 2014 participants a subset of 706 were invited to re-attend after 3 months. This was based on a preliminary 90% power analysis using the Fisher's exact method (Supplementary Table 5), which required an estimated 2% antibody positive participants and 8% antibody negative participants at enrolment returning. A steering committee decided this was appropriate ensuring all returning participants were representative of the entire enrolment cohort. Therefore, all 476 positive participants at enrolment and 230 negative participants at enrolment, randomly selected to match the study site and occupational status using a random selection algorithm, were invited. Five hundred and forty-five participants (399 IgG antibody positive participants and 146 antibody negative participants at enrolment) returned for follow up testing, Figures 1A,B.

### Sample Collection

For the ME study, venepuncture was performed on each participant utilising the site's standard blood collection method. EDTA plasma vacutainers (6 ml in total) and one 6 ml serum vacutainer were collected. Additionally, one fingerstick capillary specimen was collected from each participant. In the HCW study, each participant was required to donate only 6 ml total blood in an EDTA plasma vacutainer at each visit. To generate serum and plasma, venous blood samples were centrifuged at room temperature at 3,000 g for 15 min, aliquoted and frozen on the day of collection.

### Panbio™ COVID-19 IgG/IgM Rapid Test Device

The Panbio™ COVID-19 IgG/IgM Rapid Test Device (Fingerstick Whole Blood/Venous Whole Blood/Serum/Plasma) (Panbio™; Abbott Rapid Diagnostics Jena GmbH, Jena, Germany) assay detects IgG against the SARS-CoV-2 nucleocapsid (N) protein as well as SARS-CoV-2 IgM antibodies provided as a separate result. The clinical performance of the test device is described in the Supplementary Section. Testing was conducted according to the manufacturer's instructions for use. Briefly, samples of 20 μl (fingerstick and venous whole blood) or 10 μl (serum and plasma) were applied to the specimen well of the test device, followed by two drops (~60 μl) of buffer and a timer was started. Each ME study sample was interpreted at 10 min and again at 20 min by the same study staff member. For the HCW study, each plasma sample was interpreted at 15 min. All staff interpreting Panbio™ tests were blinded to the participants' previous exposure to SARS-CoV-2.

### Reference Testing

#### Architect™ SARS-CoV-2 IgG Test

Frozen aliquots of plasma or serum were used to conduct study reference testing, in accordance with local laboratory standard operating procedures. The primary reference test performed on the Abbott Architect i2000 chemiluminescent microparticle immunoassay (Architect) was for SARS-CoV-2 IgG (Abbott Diagnostics, IL, USA; Architect) which detects IgG against the SARS-CoV-2 nucleocapsid (N) protein. The clinical performance of the immunoassay is described in the Supplementary Section. Antibody levels ≥1.4 (manufacturer's arbitrary units; Architect Index: ratio between the sample to the internal calibrator absorbance; S/C or S/CO) were considered positive (22). The Architect Index result was used as a semi-quantitative measure of antibody positivity (3).

All 228 samples from the ME study were tested on the Architect™. Whilst in the HCW study, based on the Panbio™ high sensitivity (99.1%, 95% CI:95.3, 100.0) only the samples that gave a positive enrolment Panbio™ test reading were analysed on the Architect™. At the 3-month HCW study follow-up, all samples that were newly Panbio™ test positive as well as samples that were Panbio™ test positive at enrolment (irrespective if they gave Panbio™ negative results after 3-months) were further tested on the Architect™.

#### Roche Elecsys® Anti-SARS-CoV-2 Test

Discrepant samples with a positive Panbio™ test device reading and a negative Architect™ test reading were further analysed on the Roche Cobas e801 analyzer using the Elecsys® anti-SARS-CoV-2 assay (Elecsys® Roche Diagnostics International Ltd, Rotkreuz, Switzerland; Roche Elecsys®) according to the manufacturer's instructions. The assay detects IgG against the SARS-CoV-2 nucleocapsid (N) antigen, as well as SARS-CoV-2 IgM and IgA antibodies which are provided as a combined result (3, 9).

#### EDI™ Novel Coronavirus COVID-19 ELISA Kits

The Panbio™ COVID-19 IgG/IgM test device results for the ME study were also evaluated against the EDI™ Novel Coronavirus COVID-19 ELISA kits (Epitope Diagnostics, Inc., San Diego, USA); these consisted of separate kits designed to identify human IgG and IgM reacting to multiple epitopes of SARS-CoV-2 full length nucleocapsid (N) protein. The EDI™ Novel Coronavirus COVID-19 IgM ELISA test was the only available SARS-CoV-2 IgM reference test in the study; further discrepant IgM result resolutions were not conducted.

#### Matrix Equivalence

A matrix equivalence analysis for the SARS-CoV-2 IgG result was conducted for the Panbio™ test using fingerstick whole blood samples, venous whole blood samples and serum samples in comparison with the Panbio™ test using venous plasma samples from the same participant.

### Interpretation of the Results—Composite Reference Method

As part of the discrepant result resolution, the Panbio™ test performance for IgG was evaluated against a composite reference result, that is, Architect™ test and the Elecsys® test. The composite reference result was considered positive if either the Architect™ or Elecsys® reference test result was positive. For the HCW study, participants were considered SARS-CoV-2 antibody positive vs. negative as described in Table 1.

**Table 1:** Final SARS-CoV-2 (Covid-19) IgG antibody result status after discrepant result resolution.

**Panbio™ COVID-19 IgG/IgM test device**	**Architect™ SARS-CoV-2 IgG test**	**Roche Elecsys® anti-SARS-CoV-2 test**	**Final IgG result**
Negative	-	-	Negative
Negative	Negative	-	Negative
Negative	Positive	-	Positive
Positive	Positive	-	Positive
Positive	Negative	Positive	Positive
Positive	Negative	Negative	Negative

*Dash indicates further testing was not required*.

### Data Analysis

The software PASS v13 (Pass Software, Rijswijk, The Netherlands) was used for sample size calculation and the software SAS v9.4 (SAS, Cary, North Carolina, USA) and GraphPad prism version 9.0 (GraphPad Software LLC, California, USA) was used for statistical analyses. The study data was anonymised at source and the data analysis was performed partially by the study sponsor and the authors. For analysis of IgG levels at enrolment and persistence over three months, an Architect Index ≥1.4 was considered positive and 4 categorical levels of Architect Index were derived as quartiles of the baseline positive population. Ordinal logistic regression was conducted to characterize the relationship between baseline demographics, medical history, and COVID-19 symptoms and the four categories of Architect Index. The ordinal logistic regression assumes that the relationship between Architect Index and the subject's IgG concentration is monotonic, but not necessarily linear. To compare IgG prevalence amongst groups, one-way ANOVA or Student T test were used.

## Results

### Study Population for the ME Study

A total of 228 participants comprised of 103 males and 125 females ranging in age from 20 to 69 years were enrolled in the ME study. The participant ethnicities were White (124; 54.4%), Asian (78; 34.2%), and Black (17; 7.5%). One participant was mixed race (0.4%) and 8 participants (3.5%) did not disclose ethnicity information. One hundred twenty-three participants (53.9%) reported past COVID-19 symptoms, whereas 105 (46.1%) did not. The most common symptoms were fatigue (90; 39.5%), fever (89; 39.0%) and muscle ache (84; 36.8%). Fifteen COVID-19 IgG antibody-positive participants (6.6% of the study population) had experienced an asymptomatic SARS-CoV-2 infection. Forty-one participants (18.0%) had been hospitalized for COVID-19 illnesses. Eleven participants (4.8%) had been admitted to Intensive Care, and 3 participants (1.3%) had required invasive ventilation. Eighty-nine participants (39.0%) had a past PCR-confirmed diagnosis of COVID-19. For 87/89, the date of the PCR result was available; the positive PCR results had been obtained 13–80 days prior to study enrolment (*n* = 87, mean 51 ± 14.2 days). In total, 115 of 228 participants had a positive reference test for antibodies against SARS-CoV-2 based on Architect™ or Elecsys® testing, resulting in a total SARS-CoV-2 antibody prevalence of 50.4%.

### Evaluation of the Abbott Panbio™ COVID-19 IgG/IgM Rapid Test Device

The positive percent agreement (PPA) and negative percent agreement (NPA) of the Panbio™ COVID-19 IgG test was assessed with the Architect™ SARS-CoV-2 IgG assay as the primary reference method (Table 2A). The discrepant results (Panbio™ positive / Architect™ negative samples) were resolved by the Roche Elecsys® SARS-CoV-2 assay (Table 2B), where a composite reference result consisted of the Abbott Architect™ SARS-CoV-2 IgG test and Roche Elecsys® anti-SARS-CoV-2 test and was considered positive if either the Architect™ or the Elecsys® test was positive. For samples without an Elecsys® result, the Architect™ result was the composite reference result.

**Table 2:** Panbio™ IgG Positive percent agreement (PPA) and Negative percent agreement (NPA) using a 10-minute read time with (A) the Abbott Architect™ SARS-CoV-2 IgG test as the primary reference method, (B) a composite reference method of Architect™ and Elecsys® based on discrepant result resolution of Panbio™ positive/Architect™ negative results.

	**Total**	**True** **positive**	**False** **positive**	**True** **negative**	**False** **negative**	**PPA** **(95% CI) (%)**	**NPA** **(95% CI) (%)**
**(A)**							
Fingerstick whole blood	227^*^	102	10	114	1	99.0 (94.7, 100.0)	91.9 (85.7, 96.1)
Venous whole blood	227^**^	102	13	111	1	99.0 (94.7, 100.0)	89.5 (82.7, 94.3)
Serum	228	96	9	116	7	93.2 (86.5, 97.2)	92.8 (86.8, 96.7)
Plasma	228	103	22	103	0	100.0 (96.5, 100.0)	82.4 (74.6, 88.6)
**(B)**							
Fingerstick whole blood	227^*^	108	4	108	7	93.9 (87.9, 97.5)	96.4 (91.1, 99.0)
Venous whole blood	227^**^	111	4	108	4	96.5 (91.3, 99.0)	96.4 (91.1, 99.0)
Serum	228	101	4	109	14	87.8 (80.4, 93.2)	96.5 (91.2, 99.0)
Plasma	228	114	11	102	1	99.1 (95.3, 100.0)	90.3 (83.2, 95.0)

* *One subject had no result on fingerstick capillary whole blood testing at 10 min*.

** *One subject had an invalid test result using venous blood at 10 min. Exact Clopper-Pearson method used to calculate 95% CI = 95% confidence interval*.

The IgG results demonstrated a high PPA of the Panbio™ COVID-19 IgG/IgM test in comparison with the Architect™ SARS-CoV-2 IgG test, when used with fingerstick and venous whole blood and with plasma; the NPA was lower. With serum, the Panbio™ COVID-19 IgG/IgM test PPA was lower than with other sample types. The NPA at 10 min using the composite reference result increased for all sample types. The Architect™ assay detects SARS-CoV-2 IgG antibodies only, whereas the Elecsys® result consists of a composite SARS-COV-2 IgG, IgM and IgA result. However, the participants with a positive Elecsys® reference result and a negative Architect™ reference result all had a negative EDI™ Novel Coronavirus COVID-19 IgM ELISA test result, indicating a lack of IgM influence on the final reference test result. The PPA was decreased for whole blood and serum but remained >93.9%. Several of the false negative Panbio™ COVID-19 IgG/IgM test results were obtained for participants whose COVID-19 infection had been asymptomatic. There were no significant differences between the 10- and the 20-min readings (data not shown). In the study, all participants with a positive EDI™ Novel Coronavirus COVID-19 IgM ELISA test result also had a positive SARS-C0V-2 IgG reference result on the Architect™ assay.

Additionally, a matrix equivalence analysis was conducted for the Panbio™ COVID-19 IgG/IgM test using fingerstick whole blood samples, venous whole blood samples and serum samples in comparison with the Panbio™ COVID-19 IgG/IgM test using venous plasma samples (Supplementary Table 1). The IgG test reached 95% negative agreement, but did not reach 95% positive agreement, for a Panbio™ COVID-19 IgG/IgM test fingerstick whole blood, venous whole blood or serum test result when compared with a Panbio™ plasma test result obtained from the same participant. The Panbio™ COVID-19 IgG/IgM test device results for the ME study were also evaluated against the EDI™ Novel Coronavirus COVID-19 ELISA kits, providing an accuracy of ≥84% for IgG and ≥73% for IgM (Supplementary Tables 2, 3). To show the sensitivity reference frame Supplementary Table 4 shows an agreement between the performance of Panbio™, Architect™ and Elecsys® for the ME study PCR positives.

The results obtained from the ME study provided the rationale to use plasma-based samples for the Panbio™ to conduct the Health-Care Worker study.

### Study Population for the Health-Care Worker Study

Of the 2014 healthcare workers at Barts Health NHS Trust (London, UK) enrolled into the HCW study (Table 3), between June-August 2020, a total of 2001 were evaluable. They comprised of 551 (27.5%) males, 1,449 (72.4%) females and 1 (0.05%) undisclosed gender at enrolment, with an age range of 18- to 77-years. The participants included 1292 (64.6%) frontline HCWs who had direct contact with patients within the emergency department (ED), intensive care unit (ICU) and COVID-19 wards (frontline ever), as well as 709 (35.4%) non-frontline staff who included all other clinical and non-clinical staff (frontline never). The most represented ethnic groups identified within the cohort between enrolment and the 3-month period were White (ranging 46.6–48.3%), Asian (18.7–23.6%), and Black (17.4–18.2%). There were 61 participants who self-identified as mixed race (3.0%) at enrolment, 182 declared their ethnicity as Other (9%), whilst 4 participants (0.2%) preferred not to disclose information regarding ethnicity. Further detailed breakdown of ethnic groups, their corresponding age, gender and occupational categories are listed in Supplementary Tables 6, 7A,B.

**Table 3:** Subject disposition.

	**Enrolment**		**3-month follow-up**	
	**Total (*n*)**	**% Total population**	**Total (*n*)**	**% Total population**
Enrolled Subjects:	2014	100	575	100
**Enrolled subject status**				
Evaluable	2001	99.35	545	94.8
Unevaluable	13	0.65	30	5.2
**Reasons for unevaluable**				
Withdrawal	8	0.39	30	5.2
Unable to obtain sample	5	0.25	0	0.0
**Gender distribution**				
Female	1449	72.4	392	72.0
Male	551	27.5	153	28.0
Undisclosed	1	0.1	0	0.0
**Total**	2001	100	545	100
**Age range**				
18–32	628	31.4	120	22.0
33–47	740	37.0	195	35.8
48–62	557	27.8	205	37.6
63–77	76	3.8	25	4.6
**Total**	2001	100	545	100
**Work status**				
Frontline	1292	64.6	397	72.8
Non-frontline	709	35.4	148	27.2
**Total**	2001	100	545	100
**Ethnic group**				
Asian/Asian British	472	23.59	102	18.7
Black, African, Caribbean/Black British	349	17.44	99	18.2
White	933	46.63	263	48.3
Mixed/Multiple ethnic groups	61	3.05	17	3.1
Other	182	9.05	63	11.6
Unknown	4	0.25	1	0.2
**Total**	2001	100	545	100

*Total subjects during initial enrolment and subsequent 3-month follow up with: gender, occupational status, age range and ethnicity distribution; n and % of total population*.

At the 3-month follow up (between September-November 2020), 545 subjects were evaluable consisting of 153 (28.1%) males and 392 (71.9% females) between the ages of 18–77yrs. Subject descriptor distribution at follow up was similar to that at enrolment.

Past COVID-19 symptoms were reported by 977 (48.8%) participants, whereas 1022 (51.1%) reported no symptoms and this information was not available for 2 (0.1%) participants. The most common symptoms (Supplementary Table 9) were fatigue (28.2%), headache (26.6%), fever (26.3%), aches and pains (25.4%), and cough (25%). Other commonly reported symptoms were loss of taste (22.5%), sore throat (18.8%), shortness of breath (13%) and runny nose (12.4%). Less common symptoms, reported by <10% of the study population, were diarrhoea, skin rash, conjunctivitis and loss of speech. 8.3% of participants declared to have experienced other symptoms (not specified).

### SARS-CoV-2 Specific Antibody Prevalence at Enrolment and at 3-Month Follow-Up

From the 2001 participants at enrolment, 532 were Panbio™ IgG positive. Four hundred fifty three of these participants were confirmed SARS-CoV-2 positive by Architect™, and an additional 52 participants were confirmed positive by the Elecsys® test. Twenty seven participants with a positive Panbio™ result had a negative antibody result based on the laboratory testing; these 27 participants were classified as antibody negative. Forty one Panbio™ IgG-negative samples (positive at enrolment) were analysed by Architect™; one had a positive Architect™ result. In total, 506 (25.3%) participants were determined to be antibody positive at enrolment.

At the 3-month follow-up, 545 total eligible participants returned, of whom 399 had a confirmed positive SARS-CoV-2 antibody result at enrolment and 146 were antibody negative at enrolment. At the 3-month follow-up, 278 participants in total had a final SARS-CoV-2 antibody positive result; of these 278 participants, 29 had a Panbio™ negative result and 50 had an Architect™ negative result. At the follow-up, 274/399 participants (68.7%) had retained their antibody-positivity from enrolment and 125/399 (31.3%) of those who were antibody-positive at enrolment had a negative antibody result at follow-up. 4/146 participants (2.7%) whose antibody result was negative at enrolment had seroconverted. No subject developed disease requiring hospitalisation.

Considering the total HCW cohort at enrolment (Table 4A) the highest IgG prevalence was reported for male HCWs aged between 48–62 yrs (39.6%) and 63–77 yrs (35.0%). Similarly, in females the age group with the highest IgG positivity was 48–62 yrs (32.0%) followed by 63–77 yrs (30.4%). The prevalence was higher for males than females for all age groups. To note, these age groups were arbitrarily chosen for the cohort by dividing the age range into four equal categories.

Table 4Evaluation of % IgG prevalence in relation to: (A) age and gender, (B) ethnicity and occupational role.

**Table:** 

		**Enrolment**					
	**Age range**	***N* (% of study population)**	**IgG prevalence (%)**		**Age range**	**N (% of study population)**	**IgG prevalence (%)**
**(A)**							
Female	18–32	461 (23.0)	17.4	Male	18–32	167 (8.3)	26.3
	33–47	535 (26.7)	22.1		33–47	205 (10.2)	24.4
	48–62	397 (19.8)	32.0		48–62	159 (7.9)	39.6
	63–77	56 (2.8)	30.4		63–77	20 (1.0)	35
	Total	1449	23.6		Total	551	29.8

**Table:** 

	**Enrolment**	
**Ethnic group**	**N (% study population)**	**IgG prevalence (%)**
**(B)**		
Asian/Asian British	472 (23.6)	25
Black, African, Caribbean/Black British	349 (17.4)	33
White	933 (46.6)	21
Mixed/Multiple ethnic groups	61 (3.0)	23
Unknown	4 (0.2)	25
Other	182 (9.0)	35
**COVID-19 occupational exposure**		
Frontline worker	1292(64.6)	28.3
Non-frontline worker	709 (35.4)	19.9

*Results are based upon all three assays Panbio™, Architect™ and Elecsys® as described in study design*.

Among the self-assigned ethnic groups, those of Asian and Black ethnicity within the HCW study showed the highest prevalence of COVID-19 antibody positivity; the lowest antibody prevalence was observed in the White ethnic cohort (Table 4B and Supplementary Table 7B).

When categorising the participants according to their professional roles (Table 4B) IgG prevalence was higher amongst frontline workers (28.3%) compared to non-frontline workers (19.9%).

### SARS-CoV-2 Specific Antibody Levels at Enrolment

Confirmed IgG positive results using the Architect Index were grouped into four separate levels:1.4–2.65, 2.66–4.16, 4.17–5.79, and ≥5.79, based on the quartiles of their distribution. Looking at the association between age groups and the categorical level of the Architect Index, participants in the age groups 48–62 yrs, and 63–77yrs were more likely to have a higher Architect Index compared to participants in the group 18–32 yrs (OR = 2.422 and 3.034 respectively via ordinal logistic regression) (Table 5 and Supplementary Tables 8A–C). No significant differences were observed between the different genders and occupational status, however, a significant difference was observed between the different ethnic groups. Those identifying as Asian, Black, Mixed or of Other ethinicity were more likely to exhibit a higher Architect Index compared to participants of White ethnicity (OR = 1.881, 1.451, 1.166, and 1.418, respectively), where Asian ethnicity was statistically significant.

**Table 5:** Relationships between Architect Index levels catergorised within age, gender, ethnicity and occupational roles.

**Odds ratio estimates**			
**Effect**	**Point estimate**	**95% Wald confidence limits**	
**Age:**			
Age group 33–47 vs. 18–32	1.538	0.970	2.439
Age group 48–62 vs. 18–32	2.422	1.536	3.820
Age group 63–77 vs. 18–32	3.034	1.331	6.914
Gender			
Male vs. female	0.945	0.658	1.355
**Ethnicity**			
Asian vs. white	1.881	1.209	2.927
Black vs. white	1.451	0.930	2.262
Mixed vs. white	1.166	0.432	3.142
Other vs. white	1.418	0.829	2.424
**Occupational status**			
Frontline vs. non-frontline	1.209	0.834	1.753

### Past PCR Diagnosis, Symptoms, and Co-morbidities

Fifty four participants (2.7%) had a past PCR-confirmed diagnosis of COVID-19. 18.6% (94) of COVID-19 IgG antibody-positive participants had experienced asymptomatic SARS-CoV-2 infection at enrolment whilst 411 participants (81.2%) experienced associated symptoms. Information regarding symptoms was missing from 1 participant (0.2%).

Participants' medical history was also analysed, taking into account past hospitalisations and comorbidities (Supplementary Table 9). The association of co-morbidities and categorical level of the Architect Index (Table 6) illustrates that participants with hypertension were more likely to have a higher Architect Index value, compared to normotensive participants (OR = 2.128 via ordinal logistic regression). Individuals with obesity or diabetes also showed higher Architect Index values, however, this did not reach statistical significance. An ordinal logistic regression model showed no significant interactions between age, ethnicity, hypertension and COVID-19 symptoms (Table 7D).

**Table 6:** The association of co-morbidities and categorical level of the Architect Index.

**Odds ratio estimates**			
**Effect**	**Point estimate**	**95% Wald confidence limits**	
Diabetes—yes vs. no	0.974	0.491	1.932
Hypertension—yes vs. no	2.128	1.323	3.424
Respiratory illness—yes vs. no	1.093	0.586	2.038
Obesity—yes vs. no	1.748	0.584	5.227
Coronary illness—yes vs. no	1.010	0.303	3.373

Table 7Persistence and decline of SAR-CoV-2 antibody status over the 3-month study interval.

**Table:** 

**Enrolment** **IgG status**	***N***	**3-month follow up IgG status**	***N* (% of enrolment)**
**Followed-up participants (*****N*** **= 545)**			
**(A)**			
IgG positive	399	IgG positive	274 (68.7%)
		IgG negative	125 (31.3%)
IgG negative	146	IgG positive	4 (2.7%)
		IgG negative	142 (97.3%)

**Table:** 

	**Confirmed IGG positive**				
	**Enrolment (*****N*** **= 399)**	**Month 3 (*****N*** **= 274)**	**% Persistence**	***P*****-value of persistence**	**% Decline**
**(B)**					
**Age**					
18–32	85	47	55.3	<0.0001	44.7
33–47	139	84	60.4		39.6
48–62	156	125	80.1		19.9
63–77	19	18	94.7		5.3
**Gender**					
Male	121	77	63.6	0.1525	36.4
Female	278	197	70.9		29.1
**Occupational role**					
Frontline	291	198	68	0.6558	32
Non-Frontline	108	76	70.4		29.6
**Ethnicity**					
Asian	81	57	70.4	0.0055	29.6
Black	82	67	81.7		18.3
White	171	102	59.6		40.4
Mixed	13	11	84.6		15.4
Other	51	36	70.6		29.4
Missing	1	1	100		-

**Table:** 

	**Confirmed IGG positive**				
	**Enrolment (*****N*** **= 399)**	**Month 3 (*****N*** **= 274)**	**% Persistence**	***P*****-value of persistence**	**% Decline**
**(C)**					
**Medical History**					
**Cardiovascular conditions**					
No	392	267	68.1	0.0714	21.9
Yes	7	7	100		0
**Diabetes**					
No	376	257	68.4	0.5767	31.4
Yes	23	17	73.9		26.1
**Hypertension**					
No	346	225	65.0	<0.0001	35
Yes	53	49	92.5		7.5
**Obesity**					
No	391	266	68.0	0.0536	32
Yes	8	8	100		0
**Respiratory disorders**					
No	364	253	69.5	0.2469	30.5
Yes	35	21	60.0		40

**Table:** 

**Effect**	**Point estimate**	**95% Wald confidence limits**	
**(D)**			
**Odds ratio estimates**			
Age group: 33–47 vs. 18–32	1.519	0.958	2.409
Age group: 48–62 vs. 18–32	2.209	1.378	3.539
Age group: 63–77 vs. 18–32	2.903	1.246	6.765
Ethnic group: Asian vs. White	1.851	1.190	2.882
Ethnic group: Black vs. White	1.547	0.984	2.432
Ethnic group: Mixed vs. White	1.138	0.424	3.054
Ethnic group: Other vs. White	1.431	0.825	2.483
Hypertension: Yes vs. No	1.494	0.895	2.494
Covid Symptoms: Yes vs. No	1.869	1.260	2.771

*(A) Changes in the total follow-up population, (B) changes for age (multiple comparisons: 33–47 vs. 18–32 p = 0.4489; 48–62 vs. 18–32 p < 0.0001; 63–77 vs. 18–32 p = 0.0013; 48–62 vs. 33–47 p = 0.0002, 63–77 vs. 33–47 p = 0.0034), gender, occupational roles, ethnicities (multiple comparisons: Black vs. white p = 0.0005), (C) medical history. P-values < 0.05 were considered significant and (D) Odd ratio estimates from the ordinal logistical regression for age, ethnicity, hypertension and COVID-19 symptoms*.

The relationship between self-reported COVID-19 symptoms and the categorical level of the Architect Index was analysed by ordinal logistic regression. Participants reporting at least one of the possible COVID-19 defining symptoms by Public Health England (PHE) (new continuous cough, temperature ≥37.8°C, anosmia or ageusia) were more likely (OR = 1.869) to have higher Architect readings than those who did not report COVID-19 specific symptoms (Table 7D). From the 977 participants with self-reported symptoms, 542 (55.5%) were confirmed antibody negative.

An association between the days of duration of COVID-19 symptoms and the Architect Index was evaluated by using Ordinal Logistic Regression. Symptom duration of 11–15 days had greater probability (OR = 2.024) of a higher Architect Index when compared to symptom duration of 7 days or less. Symptom duration of 16 days or more had a higher Architect Index (OR = 2.29) when compared to symptom duration of 7 days or less (Supplementary Table 10).

### Difference in SARS CoV-2 Antibody Status and Levels Over a 3-Month Interval

**Figure 2:**
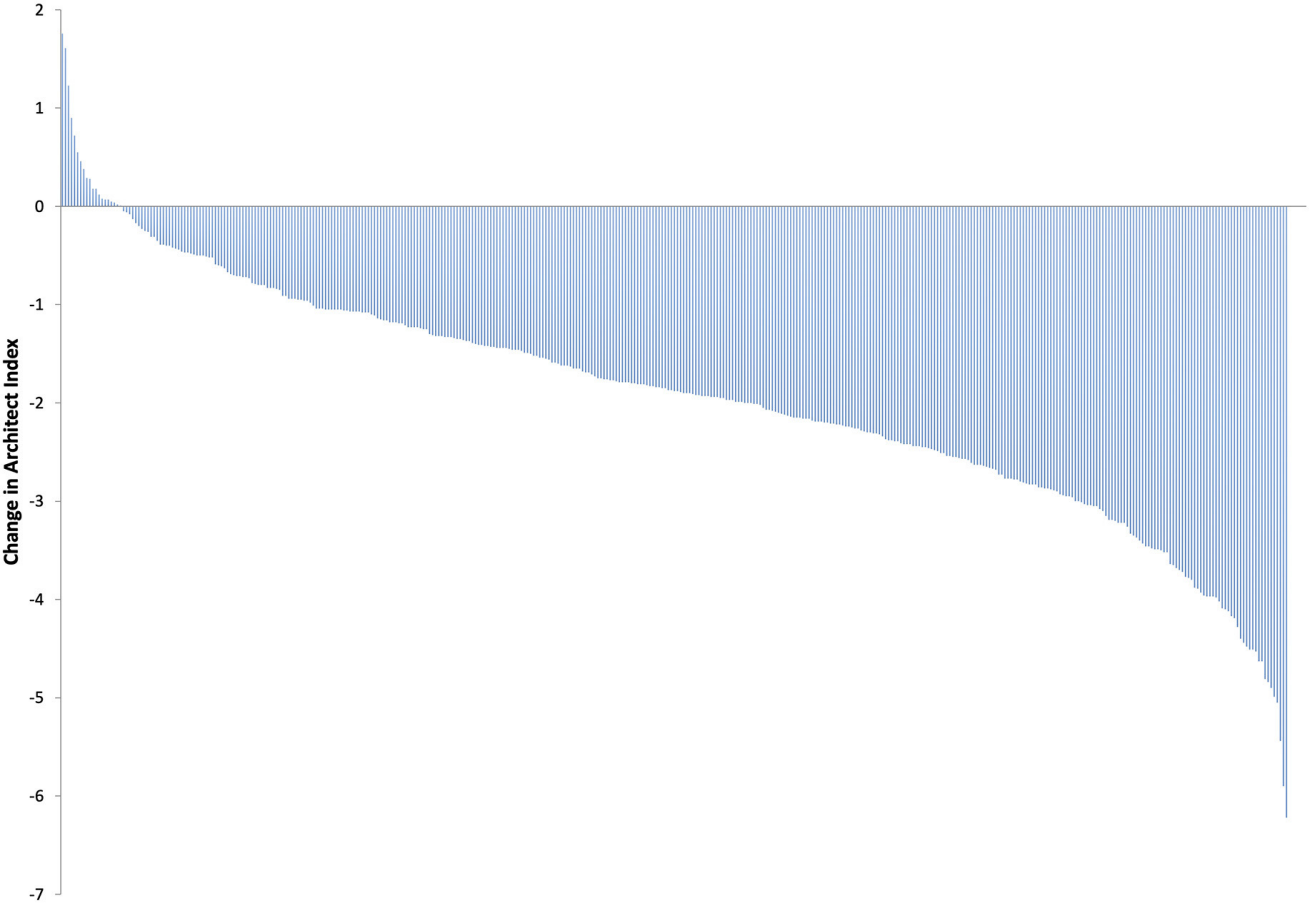
Change in Architect Index readings from baseline to 3-months in all SARS-CoV-2 IgG positive participants at enrolment.

During the 3-month study time course, 274/399 (68.7%) of the follow-up participants remained IgG positive; 3 (0.7%) had a higher Architect Index than at enrolment. Two of these participants reported at least one PHE possible COVID-19 defining symptom during the follow-up time-course. One hundred and twenty five (31.3%) participants converted from IgG positive to IgG negative whilst 4/146 antibody negative participants seroconverted to IgG positive (2.7%). The remaining 142 (97.3%) did not develop antibodies (Table 7A). When considering the overall antibody dynamics of SARS-CoV-2 IgG positive participants, a decline in the Architect Index readings was observed within the 3-month study period (Figure 2).

**Figure 3:**
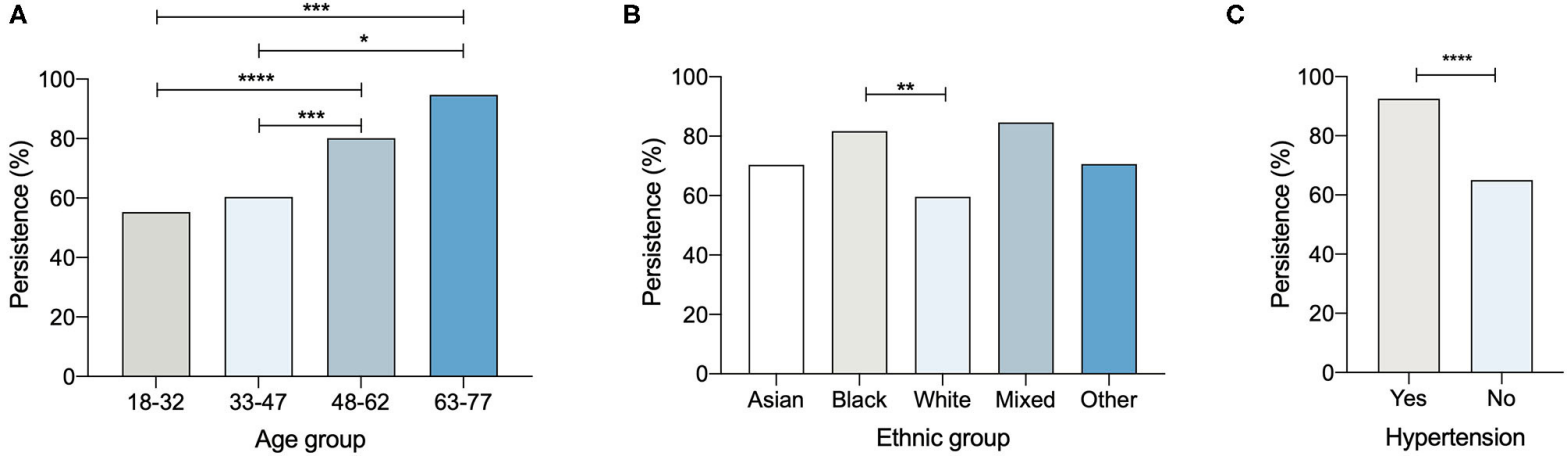
Percentage persistence of COVID-19 antibody positivity and **(A)** age, **(B)** ethnic group **(C)** hypertension (*p < 0.05; **p < 0.01; ***p < 0.005; ****p < 0.001).

To establish any relationships between the 125 participants whose IgG declined to undetectable levels after a 3-month interval, the subjects were analysed according to their ethnic groups or categorised according to work status, age, or medical history (Table 7B and Figure 3).

The persistence of SARS-CoV-2 IgG at 3-months was assessed by classifying subjects as persistently positive if Architect Index remained ≥1.4 and negative if Architect Index dropped below 1.4 (Table 8). As expected, participants with the lowest category of positive Architect Index (1.40 to 2.65) at enrolment had the lowest probability of remaining positive at 3 months (11.4%) whilst participants with higher levels of Architect Indices at enrolment (>4.16) had the highest probabilities of remaining positive at 3 months (70.2–78.6%) (Table 8).

**Table 8:** Persistence of IgG positivity after 3-months according to enrolment SARS-CoV-2 IgG levels.

**Architect index range**	**IgG positives at enrolment (*n*)**	**IgG status after 3 months**			
		**Positive (*n*)**	**Negative (*n*)**	**Non-follow up at 3 months (*n*)**	**Positivity rate**
1.4–2.65	114	13	80	21	11.4%
2.65–4.16	114	43	52	19	37.7%
4.16–5.79	114	80	11	23	70.2%
>5.79	112	88	2	22	78.6%

*n, number of participants*.

## Discussion

Since March 2020, UK hospitals have screened healthcare workers given their high exposure to SARS-CoV-2, as previously highlighted (23, 24). Nosocomial transmission has been an important amplifier in epidemics of both SARS and Middle East respiratory syndrome (25). Therefore, the rationale for hospital trusts has been to maintain the health and welfare of staff, to enable rapid identification and isolation of infected healthcare workers resulting in the protection of vulnerable patients (26) and the wider community. Over time it has become evident that the spectrum of COVID-19 symptoms is broad, ranging from asymptomatic cases, pauci-symptomatic (subclinical), pre-symptomatic (go on to develop symptoms later), or post-infection (viral RNA still detectable from a previous infection) (27).

Many HCW's remain asymptomatic (17–20%) (28–30) and modelling data indicates screening could reduce transmission by 16–23% (31), underscoring the need for widespread screening programmes of this population.

Whilst recent studies of HCWs report on the longitudinal evaluation of SARS-CoV-2 antibodies (32–35), we utilise a hospital setting to screen the performance of the Panbio™ COVID-19 IgG/IgM rapid test. Different from molecular testing, detection of a humoral immune response to the virus is an indirect marker of infection and provides a long-lasting measure of SARS-CoV-2 infection (36). As complementary diagnostic tests, they can confirm infection in symptomatic patients with high clinical suspicion who present late after illness onset, when the sensitivity of nucleic acid detection is lower. Indeed, negative antibody results of PCR positive individuals is not sufficient to exclude infection (false positivity of the molecular test) as antibody levels may be undetectable if the serologic test is performed too early (6–9) or if subjects are immunocompromised. Alternatively, absence of (or discordant) antibody responses may be due to effective clearance of infection mediated by T cells (37–39).

Robust serological tests have also demonstrated added value in epidemiological investigations for contact tracing, linking clusters of cases retrospectively (40), and determining the prevalence of the infection in high-risk categories such as HCWs or care home residents and staff (41–43).

We report a high concordance between the rapid Panbio™ COVID-19 IgG/IgM rapid test and the laboratory-based Architect™ and the Elecsys® tests indicating this may be a useful POC test for coronavirus antibody detection. However, the value of the IgM test was limited. The highest positive percent agreement (PPA) for the Panbio™ test was obtained using plasma samples (PPA 99.1%, CI:95.3, 100.0). Therefore, we analysed plasma from HCWs using the Panbio™ assay for qualitative analysis and confirmed positive results with the semi-quantitative Architect™ SARS-CoV-2 IgG assay.

We report the serologic status of 2001 hospital staff members at recruitment between June-August 2020, and 545/2001 who were longitudinally sampled at a 3-month interval from their first sampling time-point (September-November 2020). Our heterogeneous cohort reflects the unique ethnic diversity of Barts NHS Trust staff in East London, UK. From our study cohort of 2001 HCWs, we observed 506 SARS-CoV-2 (anti-N) IgG positive participants (25.3%) at enrolment. Fifty four participants (2.69% of the total cohort) had a PCR confirmed infection with a variable range of Architect Index values.

Several published studies (22, 42–44) show a varying association between age and IgG prevalence; we report higher IgG prevalence in older age groups of 48–62 yrs and 63–77 yrs, irrespective of gender. We also observed IgG prevalence was higher among males compared to females, which correlates with initial findings that linked gender-bias as a risk factor (45). Gender has been shown not to play a role in infection rates (32), but our findings could suggest gender equitable solutions are required for management of COVID-19 prevention.

Asian, Black and Other ethnicities reported higher IgG prevelance compared to White participants in our cohort. However, only the Asian ethnic group was statistically significant. This is a particularly important observation as Black and Asian ethnicities are at a higher risk of severe disease (45–48). It should be noted that our study did not account for mediators such as socioeconomic inequalities that may link ethnicity to antibody response.

Although reporting IgG concentrations would have been ideal, there was no opportunity to do this at the time the study was conducted. While the Architect Index is intended for the qualitative detection of IgG, the reported units of Architect Index are related to IgG concentration by a monotonic calibration curve. It is a limitation of the study that the calibration curve was not determined at the time of the measurements. However, by segmenting the population into quartiles of the Architect Index the relationship between IgG concentration and baseline characteristics can be explored. Complementing previous studies earlier in the course of the pandemic, which identified certain co-morbidities as risk factors for more severe manifestations of COVID-19 (45, 49–51), we report that hypertension is associated with higher Architect Indices (OR = 2.128). In parallel to the higher IgG readings at enrolment within the Black and Asian ethnic groups as well as the older participants and those with hypertension, at the 3-month follow-up these groups showed a smaller decrease in antibody positivity compared to the reference groups (White, 18–32 yrs and normotensive, respectively). Additionally, IgG prevalence at enrolment was higher among frontline workers (28.3%) compared to non-frontline workers (19.9%) with a similar decline rate observed after 3 months.

We observed that participants who declared COVID-19 defining symptoms (as specified by the PHE) had higher Architect Index readings than those with other symptoms (e.g., headache, runny nose, diarrhoea) or who were asymptomatic (OR = 1.869). Additionally, we observed that participants with a symptom duration of more than 11 days were twice as likely to have elevated Architect Indices. This is consistent with findings which suggest the presence and duration of symptoms along with disease severity were more likely to influence the development of an adequate and persistent serum immune response (51).

Among the 545 participants included in the 3-month follow-up analysis, 3 out of the 399 IgG positive subjects (0.7%) at enrolment were observed to have higher Architect Index values at follow-up, which could be explained by reinfection or the timing of the first sample, possibly taken whilst immunity was building. To date, there is limited evidence of reinfection by SARS-CoV-2, although it is generally assumed that reinfections by coronaviruses occur (52–54). In our study, a reinfection could be an explanation for 2 of the 3 subjects, as they reported COVID-19 defining symptoms a week prior to follow-up testing. The third participant only reported symptoms before enrolment in March 2020. Notably, only 4 out of the 146 antibody negative participants at enrolment (2.7%) became positive after 3 months. Similar to the much larger SIREN study (55), which recruited nationally, our local cohort data suggests that those that had a known previous infection did not become re-infected during the study period. However, we were not able to determine an association between previous infections and lower risks of new infections due to the lack of PCR information. Additionally, the low rates of seroconversion (2.7%), indicative of new infections during the September-November period (although not confirmed by PCR), may have resulted from improved workplace containment practices.

The most striking finding of this study is that 31.3% of the IgG positive participants at enrolment were found to have a negative result after 3 months, furthermore, most participants (94%) experienced a decline in their IgG antibody readings, including those with high initial Architect Indices. Our study confirms data reported by the Centre for Disease Control and Prevention, where 94% of HCWs experienced a fall in IgG levels within 60 days, with 28% sero-reverting (56).

In our study we measured SARS-CoV-2 anti-N IgG using the Abbott Architect^TM^, which has been reported to correlate with neutralizing antibody titres (57, 58). However, we recognise that this does not provide an entire picture of the anti-SARS-CoV-2 humoral immunity in the analysed participants. Anti-N and anti-S IgG have been shown to present different kinetics; anti-N IgG, detected using different automated assays, appears earlier in infection but disappears faster than anti-S IgG (59).

SARS-CoV-2 anti-N IgG and anti-SARS-CoV-2 neutralising antibodies have shown a similar decline over time (60) hence we consider it plausible that our findings can be extrapolated to describe antibody production in general. Further studies regarding total anti-SARS-CoV-2 IgG kinetics are necessary to address this knowledge gap.

The humoral immune response against human endemic coronaviruses is known to wane over time (allowing potential reinfections after 6–12 months) (61), while specific antibodies against SARS-CoV-1 have been detected for up to 17 years post infection (62, 63). Recent studies have shown different proportions of sero-reversion in SARS-CoV-2 infected individuals, determined by anti-Spike and anti-Nucleocapsid assays (34, 35). The magnitude of the neutralising antibody (anti-Spike1 nAb) has been shown to be dependent on the severity of infection resulting in individuals with mild disease having modest nAb titers having an undetectable neutralising response 50 days after the onset of symptoms (64). Whereas other studies looking at mild-moderate disease have shown detectable levels for up to 5 months (65).

In this study, we report on the Panbio^TM^ as a point of care test using the Architect assay as a semi-quantitative confirmatory assay, thereby defining a potential role of the Panbio^TM^ in epidemiological sero-surveillance or the assistance in management of COVID-19 in the future. HCW subjects with pre-defined risk factors for serious COVID-19 illness demonstrated greater prevalence, higher levels and greater persistence of SARS-CoV-2 IgG antibody than those deemed low risk. It is accepted that we will require an arsenal of tools at our disposal to diagnose early, manage and contain community outbreaks. In line with this, we envisage that the Panbio^TM^ will be incorporated as part of a battery of tests to provide diagnostic information and facilitate interventions, as it will allow the distinction between antibody production induced by natural infection and vaccination, the latter inducing anti-S, but not anti-N antibody production. Future studies should focus on better understanding antibody prevalence and persistence especially in high-risk populations aided with POC testing methods in the COVID-19 diagnostic algorithm.

## Data Availability Statement

The raw data supporting the conclusions of this article will be made available by the authors, without undue reservation.

## Ethics Statement

The studies involving human participants were reviewed and approved by South Central-Berkshire Research Ethics Committee ref: 20/SC/0191, ISRCTN60400862. London-Camden & Kings Cross Research Ethics Committee, ref 20/HRA/2675, ISRCTN15634328. The patients/participants provided their written informed consent to participate in this study.

## Author Contributions

GF and PK: study concept and design and study supervision. NC, CU, and UG: acquisition of data. KD, NC, CU, and VS: data analysis. KD, NC, CU, VS, and UG: statistical analysis. KD, DL, RB, MN, ST, MH, TC-M, and UG: admin/technical and material support. PK: ethics. NC, CU, and VS: drafting of manuscript. UG, KD, GF, and PK: critical review of manuscript. All authors contributed to the article and approved the submitted version.

## Conflict of Interest

The authors declare that this study received funding from Abbott Rapid Diagnostics. The funder had the following involvement in the study: study concept, data analysis, statistical analysis, admin/technical support, ethics and critical review of manuscript, Electronic Data Capture database development and management, statistical programming.
