# Supplementary material for: Disparities of SARS-CoV-2 Nucleoprotein-Specific IgG in Healthcare Workers in East London, UK
Source: Front Med (Lausanne). 2021 Apr 27;8:642723. doi: 10.3389/fmed.2021.642723 (PMC8111172; doi:10.3389/fmed.2021.642723)
Supplement: Supplementary file 1 [file Data_Sheet_2.docx]

**SUPPLEMENTARY MATERIAL**

**ME study Eligibility**

**Participant inclusion criteria**

1. Aged 18 years or older

2. Known to have been infected with SARS-CoV-2, or who have not been infected with SARS-CoV-2

3. Agrees to complete all aspects of the study

**Participant type:** Mixed

**Age group:** Adult

**Gender:** Both

**Target number of participants:** The intended study population will include at least 103 evaluable participants who are known to have been infected with SARS-CoV-2, and at least 103 evaluable participants who have not been infected with SARS-CoV-2.

**Participant exclusion criteria**

1. Belongs to a study group that has been filled

2. Has already participated in this study on a previous occasion

3. Is enrolled in a study to evaluate a new drug

4. Unable or unwilling to provide informed consent

5. Is a vulnerable person as deemed unfit for the study by the Principal Investigator

**HCW study Eligibility**

**Participant inclusion criteria**

1. Aged ≥18 years

2. Agrees to complete all aspects of the study

**Participant type:** Mixed

**Age group:** Adult

**Gender:** Both

**Target number of participants: Up to 2000 healthcare workers**

**Participant exclusion criteria**

1. Previously participated in this study on a previous occasion

2. Unable or unwilling to provide informed consent

3. Is a vulnerable person as deemed unfit for the study by the Principal Investigator

4. Current symptoms of COVID-19 or has had COVID-19 symptoms within the last 14 days

**Clinical performance of Panbio™ COVID-19 IgG/IgM test**

The clinical performance of the Panbio™ COVID-19 IgG/IgM test had been determined by testing 89 positive frozen EDTA and heparin plasma specimens (confirmed positive with a leading commercial PCR test) and 250 negative frozen EDTA plasma specimens collected prior to September 2019. The overall study results for IgG and IgM combined were as follows: Sensitivity 97.8% (95 % CI; 92.1-99.7), specificity 92.8% (88.,9, 95.7). For IgG , the sensitivity was 86/89 (96.6%) and the specificity was 240/250 (96%). Of the positive samples, 48 were obtained 5 days or more after a positive PCR, 37 were obtained 10 days or more after a positive PCR result and for 4 samples the time since the positive PCR result was unknown (1).

**Clinical performance of Architect™ SARS-CoV-2 IgG test**

The clinical performance of the Architect™ test had been determined using 122 serum and plasma specimens, collected at different times from 31 subjects who tested positive for SARS-CoV-2 by a PCR method and who also presented with COVID-19 symptoms. The positive percent agreement (PPA) between the Architect™SARS-CoV-2 IgG assay and the PCR comparator was calculated for each specimen, including the 95% confidence interval. To estimate the negative percent agreement (NPA), 1070 serum and plasma specimens from subjects assumed to be negative for SARS-CoV-2 were tested. Of the 1070 specimens, 997 specimens were collected prior to September 2019 (pre-COVID-19 outbreak). An additional 73 specimens were collected in 2020 from subjects who were exhibiting signs of respiratory illness but tested negative for SARS-CoV-2 by a PCR method. All 1070 specimens were tested using the SARS-CoV-2 IgG assay and the NPA and the 95% CI were calculated. The PPA values were 0% (95% CI; 0.00, 60.24) at < 3 days post symptoms onset, 25% (3,19, 65.09) at 3-7 days post symptoms onset, 86.36% (65.09, 97.09) at 8-13 days post symptoms onset and 100.00% (95.89, 100.00) at ≥ 14 days post symptoms onset. The NPA was 99.63% (99.05, 99.90) for all specimens (2).

**Panbio™ COVID-19 IgG/IgM Test Performance (IgG) with the Abbott Architect™ SARS-CoV-2 IgG Test as the Primary Reference Method**

The IgG results analyses demonstrated that the PPA of the Panbio™ COVID-19 IgG/IgM test at 10 minutes was higher than 95% in comparison to the Architect™ SARS-CoV-2 IgG test when used with fingerstick, venous whole blood, and plasma. Similarly, the NPA at 10 minutes was higher than 90% with the same three sample types. On the other hand, the Panbio™ COVID-19 IgG/IgM test PPA at 10 minutes with serum was lower than 95%, and NPA was lower than 90%. There was no significant difference in the specificity and sensitivity for Panbio™ COVID-19 IgG/IgM in comparison with Architect, between all sample types at 10 and 20 minutes.

**Supplementary Table 1.** **Panbio™ COVID-19 IgG/IgM Agreement (IgG) against Panbio™ COVID-19 IgG/IgM with Plasma (IgG)**.

A matrix equivalence analysis was conducted for the Panbio™ COVID-19 IgG/IgM test using fingerstick whole blood samples, venous whole blood samples and serum samples in comparison with the Panbio™ COVID-19 IgG/IgM test using venous plasma samples. The Panbio™ COVID-19 IgG/IgM results were evaluated using the IgG result only. These results show the differences in the test performance in various matrices versus plasma, as also observed in comparison with laboratory reference testing, where the highest sensitivity and lowest specificity was observed with plasma. This is reflected in the lower positive agreement between fingerstick and venous whole blood as well as serum in comparison with plasma. No significant differences were observed between the 10- and the 20-minute results.

|  |  | **Total** | **True**  **Positive** | **False**  **Positive** | **True**  **Negative** | **False**  **Negative** | **PPA**  **(95% CI) (%)** | **NPA**  **(95% CI) (%)** | **Accuracy**  **(95% CI) (%)** |
| --- | --- | --- | --- | --- | --- | --- | --- | --- | --- |
| **Fingerstick capillary whole blood** | 10 min | 227* | 109 | 3 | 99 | 16 | 87.2 (80.0, 92.5) | 97.1 (91.6, 99.4) | 91.6 (87.2, 94.9) |
|  | 20 min | 228 | 110 | 4 | 100 | 14 | 88.7 (81.8, 93.7) | 96.2 (90.4, 98.9) | 92.1 (87.8, 95.3) |
| **Venous whole blood** | 10 min | 227** | 113 | 2 | 100 | 12 | 90.4 (83.8, 94.9) | 98.0 (93.1, 99.8) | 93.8 (89.9, 96.6) |
|  | 20 min | 228 | 114 | 3 | 101 | 10 | 91.9 (85.7, 96.1) | 97.1 (91.8, 99.4) | 94.3 (90.4, 96.9) |
| **Serum** | 10 min | 228 | 105 | 0 | 103 | 20 | 84.0 (76.4, 89.9) | 100.0 (96.5, 100.0) | 91.2 (86.8, 94.6) |
|  | 20 min | 228 | 106 | 1 | 103 | 18 | 85.5 (78.0, 91.2) | 99.0 (94.8, 100.0) | 91.7 (87.3, 94.9) |

**Supplementary Table 2. Panbio™ COVID-19 IgG/IgM test performance versus the Epitope™ IgG ELISA test*.*** Panbio™ COVID-19 IgG/IgM analyses were conducted using the Epitope™ IgG ELISA test as reference method. These analyses are applicable only for the IgG test of the Panbio™ COVID-19 IgG/IgM rapid test device.

|  |  | **Total** | **True**  **Positive** | **False**  **Positive** | **True**  **Negative** | **False**  **Negative** | **PPA**  **(95% CI) (%)** | **NPA**  **(95% CI) (%)** | **Accuracy**  **(95% CI) (%)** |
| --- | --- | --- | --- | --- | --- | --- | --- | --- | --- |
| **Fingerstick capillary**  **whole blood** | 10 min | 225 | 83 | 27 | 111 | 4 | 95.4 (88.6, 98.7) | 80.4 (72.8, 86.7) | 86.2 (81.0, 90.4) |
|  | 20 min | 226 | 85 | 27 | 112 | 2 | 97.7 (91.9, 99.7) | 80.6 (73.0, 86.8) | 87.2 (82.1, 91.2) |
| **Venous whole blood** | 10 min | 225 | 86 | 27 | 111 | 1 | 98.9 (93.8, 100.0) | 80.4 (72.8, 86.7) | 87.6 (82.5, 91.6) |
|  | 20 min | 226 | 86 | 29 | 110 | 1 | 98.9 (93.8, 100.0) | 79.1 (71.4, 85.6) | 86.7 (81.6, 90.9) |
| **Serum** | 10 min | 226 | 82 | 21 | 118 | 5 | 94.3 (87.1, 98.1) | 84.9 (77.8, 90.4) | 88.5 (83.6, 92.3) |
|  | 20 min | 226 | 82 | 23 | 116 | 5 | 94.3 (87.1, 98.1) | 83.5 (76.2, 89.2) | 87.6 (82.6, 91.6) |
| **Plasma** | 10 min | 226 | 87 | 36 | 103 | 0 | 100.0 (95.8, 100.0) | 74.1 (66.0, 81.2) | 84.1 (78.6, 88.6) |
|  | 20 min | 226 | 87 | 35 | 104 | 0 | 100.0 (95.8, 100.0) | 74.8 (66.8, 81.8) | 84.5 (79.1, 89.0) |

**Supplementary Table 3. Panbio™ COVID-19 IgG/IgM test performance versus the Epitope™ IgM ELISA test**. The IgM test in the Panbio™ COVID-19 IgG/IgM rapid test device was evaluated against the Epitope™ IgM ELISA test as reference method. There were only 10 samples that were reference-positive for IgM; these samples were also reference-positive for IgG.

|  |  | **Total** | **True**  **Positive** | **False**  **Positive** | **True**  **Negative** | **False**  **Negative** | **PPA**  **(95% CI) (%)** | **NPA**  **(95% CI) (%)** | **Accuracy**  **(95% CI) (%)** |
| --- | --- | --- | --- | --- | --- | --- | --- | --- | --- |
| **Fingerstick capillary whole blood** | 10 min | 217 | 4 | 1 | 206 | 6 | 40.0 (12.2, 73.8) | 99.5 (97.3, 100.0) | 96.8 (93.5, 98.7) |
|  | 20 min | 218 | 5 | 1 | 207 | 5 | 50.0 (18.7, 81.3) | 99.5 (97.4, 100.0) | 97.2 (94.1, 99.0) |
| **Venous whole blood** | 10 min | 217 | 3 | 3 | 204 | 7 | 30.0 (6.7, 65.2) | 98.6 (95.8, 99.7) | 95.4 (91.7, 97.8) |
|  | 20 min | 218 | 3 | 4 | 204 | 7 | 30.0 (6.7, 65.2) | 98.1 (95.1, 99.5) | 95.0 (91.2, 97.5) |
| **Serum** | 10 min | 218 | 4 | 12 | 196 | 6 | 40.0 (12.2, 73.8) | 94.2 (90.1, 97.0) | 91.7 (87.3, 95.0) |
|  | 20 min | 218 | 4 | 13 | 195 | 6 | 40.0 (12.2, 73.8) | 93.8 (89.5, 96.6) | 91.3 (86.7, 94.7) |
| **Plasma** | 10 min | 218 | 6 | 52 | 156 | 4 | 60.0 (26.2, 87.8) | 75.0 (68.5, 80.7) | 74.3 (68.0, 80.0) |
|  | 20 min | 218 | 6 | 53 | 155 | 4 | 60.0 (26.2, 87.8) | 74.5 (68.0, 80.3) | 73.9 (67.5, 79.6) |

**Supplementary Table 4.** Agreement between the performance of Panbio™ and Architect™ for the ME study participants with an available RT-PCR result before enrolment**.**

|  |  |  |  | Panbio^TM^ | | | | | | | |
| --- | --- | --- | --- | --- | --- | --- | --- | --- | --- | --- | --- |
| RT-PCR | | Architect^TM^ | | Fingerstick | | Venous blood | | Serum | | Plasma | |
|  |  | Pos | Neg | Pos | Neg | Pos | Neg | Pos | Neg | Pos | Neg |
| Pos | 87 | 80 | 7 | 84 | 3 | 85 | 2 | 79 | 8 | 86 | 1 |
| Neg | 23 | 3 | 20 | 4 | 19 | 4 | 19 | 4 | 19 | 4 | 19 |
| PPA (95% CI) (%) | | 92,0 | (86,3 -97,7) | 96,6 | (92,7-100) | 97,7 | (94,5-100) | 90,8 | (84,7-96,8) | 98,9 | (96,7-100) |
| NPA (95% CI) (%) | | 87,0 | (73,2-100) | 82,6 | (67,1-98) | 82,6 | (67,1-98) | 82,6 | (67,1-98) | 82,6 | (67,1-98) |

**Supplementary Table 5:** Analysis of Two Independent Proportions Tests. Numeric Results of Tests Based on the Difference: P1 - P2. H0: P1 - P2 ≥ 0.   H1: P1 - P2 = D1 < 0.   Test Statistic: Fisher's Exact test

|  |  |  |  | **TrtH1** | **Control** | **Difference** | **Target** |
| --- | --- | --- | --- | --- | --- | --- | --- |
| **Power** | **N1** | **N2** | **N** | **P1** | **P2** | **D1** | **Alpha** |
| 0.9012 | 200 | 300 | 500 | 0.0200 | 0.0800 | -0.0600 | 0.0500 |
| 0.9332 | 200 | 400 | 600 | 0.0200 | 0.0800 | -0.0600 | 0.0500 |

**Supplementary Table 6.** Demographic breakdown of ethnicity groups within the HCW study. Grouping titles were used according to UK government guidelines.

|  |  | **Enrolment** | | **3-month Follow-up** | |
| --- | --- | --- | --- | --- | --- |
|  |  | **Total (n)** | **%** | **Total (n)** | **%** |
| **Asian/Asian British** | Indian | 176 | 8.81 | 42 | 7.71 |
|  | Pakistani | 46 | 2.30 | 12 | 2.2 |
|  | Bangladeshi | 99 | 4.96 | 17 | 3.12 |
|  | Chinese | 29 | 1.45 | 7 | 1.28 |
|  | Any other Asian background | 122 | 6.11 | 24 | 4.4 |
| **Black, African, Caribbean/Black British** | African | 270 | 13.52 | 76 | 13.94 |
|  | Caribbean | 72 | 3.61 | 20 | 3.67 |
|  | Any other Black, African, or Caribbean background | 7 | 0.35 | 3 | 0.55 |
| **White** | English, Welsh, Scottish, Northern Irish or British | 658 | 32.95 | 193 | 35.4 |
|  | Irish | 65 | 3.25 | 19 | 3.49 |
|  | Any other White background | 210 | 10.52 | 51 | 9.36 |
| **Mixed/Multiple ethnic groups** | White and Black Caribbean | 8 | 0.40 | 1 | 0.18 |
|  | White and Black African | 6 | 0.30 | 3 | 0.55 |
|  | White and Asian | 19 | 0.95 | 1 | 0.18 |
|  | Any other Mixed or Multiple ethnic background | 28 | 1.40 | 12 | 2.2 |
| **Other** |  | 181 | 9.06 | 63 | 11.56 |
| **Unknown** |  | 5 | 0.25 | 1 | 0 |
| **Total** |  | **2001** |  | **545** |  |

**Supplementary Table 7. Distributions withing HCW cohort** A) spread of ethnicity groups, age range, gender, and occupational roles. At enrolment, the evaluable subjects (n) = 2001 and at 3-month follow up n = 545 B) IgG positives within each ethnicity and the relationship between age groups, gender and occupational roles

(A)

|  |  | **Enrolment** | | | | | | **3-month follow up** | | | | | |
| --- | --- | --- | --- | --- | --- | --- | --- | --- | --- | --- | --- | --- | --- |
| **Age Group** | **Ethnic group** | **Female (n)** | | | **Male (n)** | | | **Female (n)** | | | **Male (n)** | | |
| **(Years)** |  | Frontline | Non-Frontline | Both | Frontline | Non-Frontline | Both | Frontline | Non-Frontline | Both | Frontline | Non-Frontline | Both |
| **18-32** | **Asian/Asian British** | 57 | 42 | 17 | 29 | 20 | 9 | 10 | 4 | 0 | 4 | 1 | 1 |
| **33-47** |  | 65 | 57 | 13 | 23 | 15 | 6 | 18 | 9 | 1 | 4 | 2 | 1 |
| **48-62** |  | 40 | 28 | 8 | 18 | 7 | 4 | 15 | 10 | 2 | 13 | 1 | 1 |
| **63-77** |  | 6 | 5 | 1 | 0 | 1 | 0 | 3 | 2 | 0 | 0 | 0 | 0 |
| **Total** |  | **168** | **132** | **39** | **70** | **43** | **19** | **46** | **25** | **3** | **21** | **4** | **3** |
| **± SD** |  | 26.17 | 22.11 | 6.90 | 12.50 | 8.42 | 3.77 | 6.56 | 3.86 | 0.96 | 5.50 | 0.82 | 0.50 |
| **18-32** | **Black, African, Caribbean/Black British** | 31 | 21 | 7 | 5 | 3 | 0 | 5 | 3 | 1 | 3 | 1 | 0 |
| **33-47** |  | 51 | 36 | 20 | 15 | 10 | 4 | 16 | 6 | 6 | 5 | 0 | 0 |
| **48-62** |  | 53 | 38 | 17 | 14 | 6 | 5 | 23 | 11 | 6 | 5 | 1 | 2 |
| **63-77** |  | 2 | 3 | 3 | 2 | 0 | 3 | 0 | 1 | 1 | 1 | 0 | 2 |
| **Total** |  | **137** | **98** | **47** | **36** | **19** | **12** | **44** | **21** | **14** | **14** | **2** | **4** |
| **± SD** |  | 23.68 | 16.22 | 8.06 | 6.48 | 4.27 | 2.16 | 10.42 | 4.35 | 2.89 | 1.91 | 0.58 | 1.15 |
| **18-32** | **White** | 140 | 72 | 26 | 45 | 15 | 12 | 34 | 9 | 10 | 9 | 2 | 5 |
| **33-47** |  | 107 | 93 | 27 | 49 | 36 | 21 | 36 | 19 | 8 | 15 | 7 | 9 |
| **48-62** |  | 59 | 73 | 34 | 41 | 23 | 18 | 24 | 22 | 12 | 18 | 6 | 5 |
| **63-77** |  | 8 | 20 | 2 | 4 | 6 | 2 | 2 | 8 | 1 | 1 | 1 | 0 |
| **Total** |  | **314** | **258** | **89** | **139** | **80** | **53** | **96** | **58** | **31** | **43** | **16** | **19** |
| **± SD** |  | 57.58 | 31.20 | 13.96 | 20.76 | 12.73 | 8.38 | 15.58 | 7.05 | 4.79 | 7.50 | 2.94 | 3.69 |
| **18-32** | **Mixed/ Multiple ethnic groups** | 8 | 11 | 2 | 8 | 2 | 0 | 3 | 1 | 0 | 0 | 1 | 0 |
| **33-47** |  | 6 | 9 | 2 | 3 | 2 | 0 | 2 | 3 | 0 | 0 | 0 | 0 |
| **48-62** |  | 4 | 3 | 1 | 0 | 0 | 0 | 3 | 1 | 1 | 1 | 0 | 0 |
| **63-77** |  | 0 | 0 | 0 | 0 | 0 | 0 | 0 | 0 | 1 | 0 | 0 | 0 |
| **Total** |  | **18** | **23** | **5** | **11** | **4** | **0** | **8** | **5** | **2** | **1** | **1** | **0** |
| **± SD** |  | 3.42 | 5.12 | 0.96 | 3.77 | 1.15 | 0.00 | 1.41 | 1.26 | 0.58 | 0.50 | 0.50 | 0.00 |
| **18-32** | **Other** | 18 | 5 | 4 | 10 | 5 | 2 | 3 | 1 | 2 | 4 | 1 | 1 |
| **33-47** |  | 34 | 10 | 4 | 14 | 4 | 3 | 15 | 4 | 0 | 5 | 1 | 1 |
| **48-62** |  | 18 | 13 | 7 | 13 | 5 | 5 | 7 | 5 | 2 | 5 | 3 | 1 |
| **63-77** |  | 0 | 6 | 0 | 0 | 1 | 1 | 0 | 0 | 0 | 0 | 1 | 1 |
| **Total** |  | **70** | **34** | **15** | **37** | **15** | **11** | **25** | **10** | **4** | **14** | **6** | **4** |
| **± SD** |  | 13.89 | 3.70 | 2.87 | 6.40 | 1.89 | 1.71 | 6.50 | 2.38 | 1.15 | 2.38 | 1.00 | 0.00 |
| **18-32** | **Unknown** | 0 | 0 | 0 | 2 | 0 | 0 | 0 | 0 | 0 | 1 | 0 | 0 |
| **33-47** |  | 0 | 1 | 0 | 0 | 0 | 0 | 0 | 0 | 0 | 0 | 0 | 0 |
| **48-62** |  | 1 | 1 | 0 | 0 | 0 | 0 | 0 | 0 | 0 | 0 | 0 | 0 |
| **63-77** |  | 0 | 0 | 0 | 0 | 0 | 0 | 0 | 0 | 0 | 0 | 0 | 0 |
| **Total** |  | **1** | **2** | **0** | **2** | **0** | **0** | **0** | **0** | **0** | **1** | **0** | **0** |
| **± SD** |  | 0.50 | 0.58 | 0.00 | 1.00 | 0.00 | 0.00 | 0.00 | 0.00 | 0.00 | 0.50 | 0.00 | 0.00 |

(B)

|  | **Enrolment** | | | | | |
| --- | --- | --- | --- | --- | --- | --- |
| **Ethnic groups** | **Frontline** | | **Non-Frontline** | | **Both** | |
|  | N (% Study population) | (%) IgG prevalence | N (% Study population) | (%) IgG prevalence | N (% Study population) | (%) IgG prevalence |
| **Asian** | 238 (11.9) | 31.9 | 176 (8.7) | 19.9 | 58 (2.9) | 13.8 |
| **Female** | 168 (8.3) | 29.8 | 132 (6.6) | 21.2 | 39 (1.9) | 12.8 |
| **Male** | 70 (3.5) | 37.1 | 43 (2.1) | 16.3 | 19 (0.9) | 15.8 |
| **Black** | 173 (8.6) | 38.1 | 117 (5.8) | 23.9 | 59 (2.9) | 33.9 |
| **Female** | 137 (6.8) | 35.8 | 98 (4.9) | 24.5 | 47 (2.3) | 31.9 |
| **Male** | 36 (1.8) | 47.2 | 19 (0.9) | 21.1 | 12 (0.6) | 41.7 |
| **White** | 453 (22.6) | 23.6 | 338 (16.9) | 16.6 | 142 (7.1) | 22.5 |
| **Female** | 314 (15.7) | 20.4 | 258 (12.9) | 15.9 | 89 (4.4) | 20.2 |
| **Male** | 139 (6.9) | 30.9 | 80 (4.0) | 18.7 | 53 (2.6) | 26.4 |
| **Mixed** | 29 (1.5) | 27.6 | 27 (1.3) | 18.5 | 5 (0.2) | 20.0 |
| **Female** | 18 (0.9) | 38.9 | 23 (1.1) | 17.4 | 5 (0.2) | 20.0 |
| **Male** | 11 (0.5) | 9.1 | 4 (0.2) | 25 | 0 | -- |
| **Other** | 107 (5.3) | 32.7 | 49 (2.4) | 34.7 | 26 (1.3) | 42.3 |
| **Female** | 70 (3.5) | 31.4 | 34 (1.7) | 26.5 | 15 (0.8) | 33.3 |
| **Male** | 37  (1.8) | 35.1 | 15 (0.7) | 53.3 | 11 (0.5) | 54.5 |

**Supplementary Table 8**

**(A) Ordinal logistic regression for demographics**

- Intercept 4 is the estimated log odds for Level 4 Arch_Index versus Level 3 and Level 2 and Level 1 Arch_Index when the predictor variables are evaluated at zero.
- Intercept 3 is the estimated log odds for Level 4 and Level 3 Arch_Index versus Level 2 and Level 1 Arch_Index when the predictor variables are evaluated at zero.
- Intercept 2 is the estimated log odds for Level 4 and Level 3 and Level 2 Arch_Index versus Level 1 Arch_Index when the predictor variables are evaluated at zero.
- Co-efficient is the ordinal log-odds estimate of comparing one category to reference on Arch_index given the other predictor variables are held constant in the model.
- Odds-ratio is the proportional odds of comparing one category to reference on Arch_index given the other predictor variables are held constant in the model.
- Odds-ratio=exponential (co-efficient of logit regression)

| **Analysis of Maximum Likelihood Estimates** | | | | | | |
| --- | --- | --- | --- | --- | --- | --- |
| **Parameter** |  | **DF** | **Estimate** | **Standard Error** | **Wald Chi-Square** | **Pr > ChiSq** |
| **Intercept** | **4** | 1 | -2.1310 | 0.2801 | 57.8904 | <.0001 |
| **Intercept** | **3** | 1 | -0.9703 | 0.2660 | 13.3100 | 0.0003 |
| **Intercept** | **2** | 1 | 0.1886 | 0.2624 | 0.5167 | 0.4723 |
| **Age group** | **33-47** | 1 | 0.4306 | 0.2352 | 3.3510 | 0.0672 |
| **Age group** | **48-62** | 1 | 0.8847 | 0.2324 | 14.4927 | 0.0001 |
| **Age group** | **63-77** | 1 | 1.1099 | 0.4202 | 6.9760 | 0.0083 |
| **Ethnic group** | **Asian** | 1 | 0.6320 | 0.2256 | 7.8506 | 0.0051 |
| **Ethnic group** | **Black** | 1 | 0.3720 | 0.2267 | 2.6943 | 0.1007 |
| **Ethnic group** | **Mixed** | 1 | 0.1533 | 0.5060 | 0.0918 | 0.7619 |
| **Ethnic group** | **Other** | 1 | 0.3491 | 0.2736 | 1.6283 | 0.2019 |
| **Gender** | **M** | 1 | -0.0569 | 0.1842 | 0.0954 | 0.7574 |
| **Frontline** | **1** | 1 | 0.1901 | 0.1893 | 1.0084 | 0.3153 |

**(B) Ordinal logistic regression for medical history**

| **Analysis of Maximum Likelihood Estimates** | | | | | | |
| --- | --- | --- | --- | --- | --- | --- |
| **Parameter** |  | **DF** | **Estimate** | **Standard Error** | **Wald Chi-Square** | **Pr > ChiSq** |
| **Intercept** | **4** | 1 | -1.2663 | 0.1226 | 106.6977 | <.0001 |
| **Intercept** | **3** | 1 | -0.1393 | 0.1056 | 1.7388 | 0.1873 |
| **Intercept** | **2** | 1 | 0.9839 | 0.1166 | 71.2369 | <.0001 |
| **Diabetes (Y/N)** | **Yes** | 1 | -0.0263 | 0.3495 | 0.0057 | 0.9400 |
| **Hypertension (Y/N)** | **Yes** | 1 | 0.7553 | 0.2427 | 9.6834 | 0.0019 |
| **Respiratory illness (Y/N)** | **Yes** | 1 | 0.0887 | 0.3179 | 0.0779 | 0.7801 |
| **Obesity (Y/N)** | **Yes** | 1 | 0.5584 | 0.5590 | 0.9980 | 0.3178 |
| **Coronary illness (Y/N)** | **Yes** | 1 | 0.0101 | 0.6152 | 0.0003 | 0.9869 |

**(C) Ordinal logistic regression for Age, Ethnicity, Hypertension and COVID-19 symptom**

| **Analysis of Maximum Likelihood Estimates** | | | | | | |
| --- | --- | --- | --- | --- | --- | --- |
| **Parameter** |  | **DF** | **Estimate** | **Standard Error** | **Wald Chi-Square** | **Pr > ChiSq** |
| **Intercept** | **4** | 1 | -2.5213 | 0.2879 | 76.7014 | <.0001 |
| **Intercept** | **3** | 1 | -1.3358 | 0.2706 | 24.3644 | <.0001 |
| **Intercept** | **2** | 1 | -0.1551 | 0.2635 | 0.3462 | 0.5563 |
| **Age group** | **33-47** | 1 | 0.4181 | 0.2353 | 3.1565 | 0.0756 |
| **Age group** | **48-62** | 1 | 0.7924 | 0.2406 | 10.8481 | 0.0010 |
| **Age group** | **63-77** | 1 | 1.0658 | 0.4316 | 6.0983 | 0.0135 |
| **Ethnic group** | **Asian** | 1 | 0.6160 | 0.2257 | 7.4472 | 0.0064 |
| **Ethnic group** | **Black** | 1 | 0.4361 | 0.2309 | 3.5680 | 0.0589 |
| **Ethnic group** | **Mixed** | 1 | 0.1295 | 0.5035 | 0.0662 | 0.7970 |
| **Ethnic group** | **Other** | 1 | 0.3585 | 0.2811 | 1.6271 | 0.2021 |
| **Hypertension (Y/N)** | **Yes** | 1 | 0.4013 | 0.2616 | 2.3527 | 0.1251 |
| **Covid-19 symptoms (Y/N)** | **Yes** | 1 | 0.6253 | 0.2010 | 9.6830 | 0.0019 |

**Supplementary Table 9**. Medical history of evaluable subjects. a) Symptoms reported b) self-reported medical conditions N= number of subjects

|  | **Evaluable subjects at enrolment** | | |
| --- | --- | --- | --- |
|  | **Yes** | **No** | **Missing** |
| **Symptom** | 977 (48.8%) | 1022 (51.1%) | 2 (0.1%) |
| **Headache** | 533 (26.6%) | 1466 (73.3%) | 2(0.1%) |
| **Aches and pains** | 508 (25.4%) | 1491 (74.5%) | 2 (0.1%) |
| **Conjunctivitis** | 36 (1.8%) | 1963 (98.1%) | 2 (0.1%) |
| **Cough** | 500 (25.0%) | 1499 (74.9%) | 2 (0.1%) |
| **Diarrhoea** | 140 (7.0%) | 1859 (92.9%) | 2 (0.1%) |
| **Fever** | 527 (26.3%) | 1472 (73.6%) | 2 (0.1%) |
| **Runny nose** | 248 (12.4%) | 1751 (87.5%) | 2 (0.1%) |
| **Rash on skin** | 38 (1.9%) | 1961 (98.0%) | 2 (0.1%) |
| **Shortness of breath** | 260 (13.0%) | 1739 (86.9%) | 2 (0.1%) |
| **Loss of speech** | 20 (1.0%) | 1979 (98.9%) | 2 (0.1%) |
| **Loss of taste** | 450 (22.5%) | 1549 (77.4%) | 2 (0.1%) |
| **Sore throat** | 376 (18.8%) | 1623 (81.1%) | 2 (0.1%) |
| **Tiredness** | 564 (28.2%) | 1435 (71.7%) | 2 (0.1%) |
| **Other** | 166 (8.3%) | 1833 (91.6%) | 2 (0.1%) |

(A)

(B)

| **Evaluable Subjects (n=2001)** | | | |
| --- | --- | --- | --- |
|  | **Yes** | No | **Missing (N/A)** |
| Hospital | 61 (3.0%) | 1938 (96.9%) | 2 (0.1%) |
| ICU | 0 (0%) | 61 (3.0%) | 1940 (97.0%) |
| Ventilator | 0 (0%) | 61 (3.0%) | 1940 (97.0%) |
| Diabetic | 82 (4.1%) | 1917 (95.8%) | 2 (0.1%) |
| Hypertension | 174 (8.7%) | 1825 (91.2%) | 2 (0.1%) |
| HIV | 7 (0.3%) | 1992 (99.6%) | 2 (0.1%) |
| Hepatitis B | 2 (0.1%) | 1997 (99.8%) | 2 (0.1%) |
| Hepatitis C | 1 (0.0%) | 1998 (99.9%) | 2 (0.1%) |
| Respiratory | 158 (7.9%) | 1841 (92.0%) | 2 (0.1%) |
| Immunocompromised | 17 (0.8%) | 1982 (99.1%) | 2 (0.1%) |
| Severe obese | 41 (2.0%) | 1958 (97.9%) | 2 (0.1%) |
| Cardiac Disease | 29 (1.4%) | 1970 (98.5%) | 2 (0.1%) |

**Supplementary Table 10.** Ordinal logistic regression for symptom duration (days) from onset

| **Odds Ratio Estimates** | | | |
| --- | --- | --- | --- |
| **Effect** | **Point Estimate** | **95% Wald Confidence Limits** | |
| **Symptoms - days 8-10 vs ≤ 7** | 1.127 | 0.611 | 2.079 |
| **Symptoms – days 11-15 vs ≤ 7** | 2.024 | 1.202 | 3.409 |
| **Symptoms – days ≥ 16 vs ≤ 7** | 2.292 | 1.440 | 3.647 |

**References**

1. Abbott, Panbio^TM^ COVID-19 IgG/IgM Rapid Test Device (Fingerstick Whole Blood/ Venous Whole Blood/Serum/Plasma) Instructions for Use, REF ICO-T402 / ICOT40203, 2020.
2. Abbott ARCHITECT^TM^ SARS-CoV-2 IgG Instructions for Use. H14806R01. April 2020.
