# Supplementary material for: Disparities of SARS-CoV-2 Nucleoprotein-Specific IgG in Healthcare Workers in East London, UK
Source: Front Med (Lausanne). 2021 Apr 27;8:642723. doi: 10.3389/fmed.2021.642723 (PMC8111172; doi:10.3389/fmed.2021.642723)
Supplement: Supplementary file 2 [file Data_Sheet_1.docx]

Detection of SARS-CoV-2 antibody reveals past infection, permitting the evaluation of potential protection from re-infection. We analysed 2001 healthcare workers from a large London NHS Trust, either in direct contact with COVID-19 patients (frontline) or non-frontline, reflecting the unique population of East London, one of the most ethnically diverse reported on to-date. The Abbott Panbio™ rapid test and the Abbott Architect™ assay were utilised. 545 participants underwent repeat analysis at 3-months, at which time Architect Index values had declined in the majority of subjects, including those with high levels at enrolment, and 31.3% of positive participants had become negative. IgG prevalence was higher in all age-groups for males compared to females. Asian, Black and other ethnicities reported higher prevelance than White subjects. These groups were also more likely to remain antibody positive. Hypertension was associated with higher Architect readings. Antibody prevalence at enrolment was higher amongst frontline workers compared to non-frontline, but there was no difference in antibody decline at 3-months. Our results suggest the Panbio™ will be incorporated as a battery of critical tests to provide greatest diagnostic certainty and to facilitate early interventions for COVID-19. A better understanding of antibody prevalence and persistence in high-risk populations is needed.
